# Supplementary figures and images for: The type 3 secretion system requires actin polymerization to open translocon pores
Source: PLoS Pathog. 2021 Sep 9;17(9):e1009932. doi: 10.1371/journal.ppat.1009932 (PMC8454972; doi:10.1371/journal.ppat.1009932)

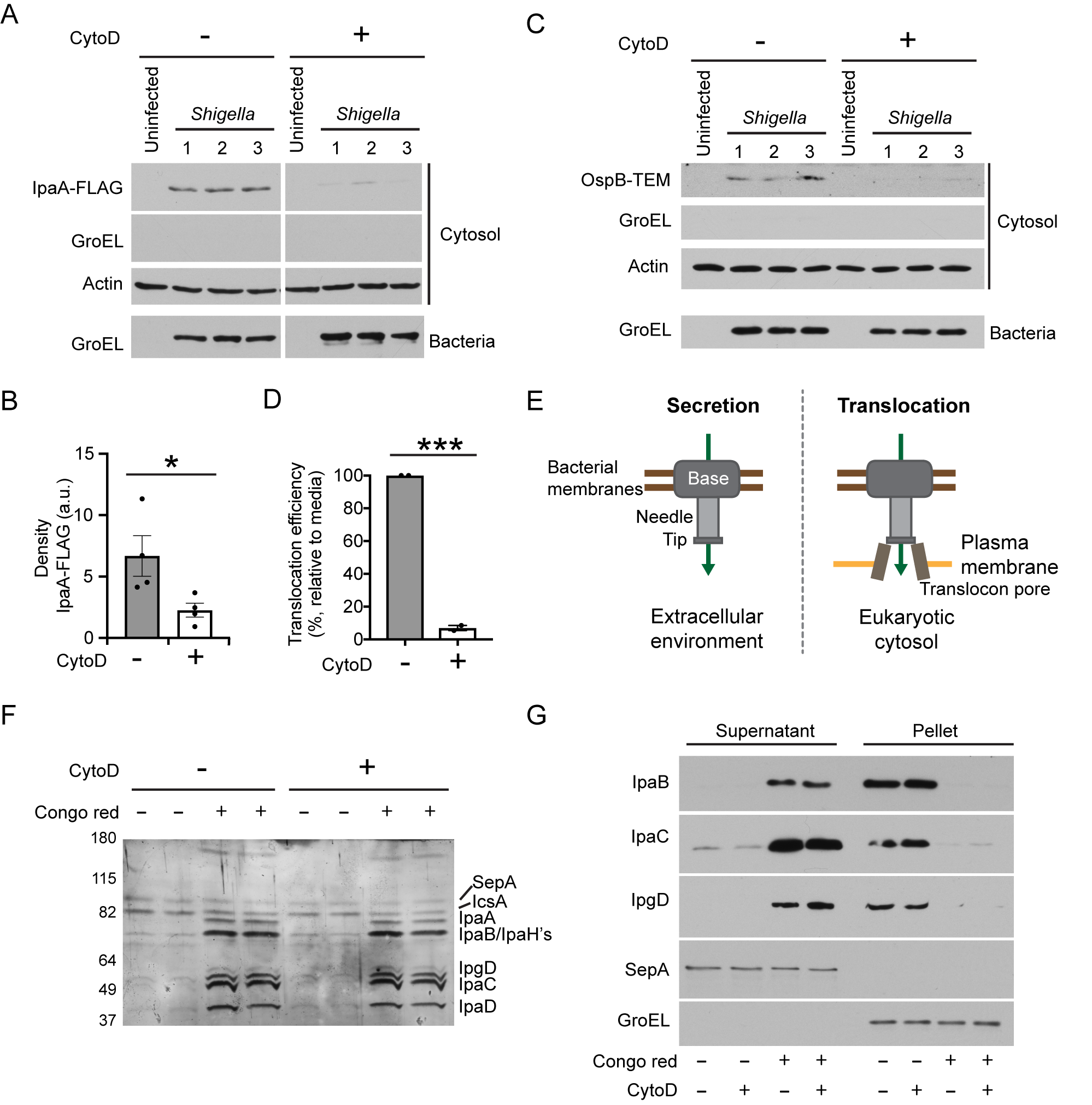

Supplement: S1 Fig — (A-D) S. flexneri translocation of the FLAG-tagged type 3 effector IpaA and the TEM β-lactamase-tagged type 3 effector OspB into HeLa cells requires actin polymerization. (A and C) Representative western blots of cytosolic FLAG-tagged IpaA (A) or TEM-tagged OspB (C) in S. flexneri infected HeLa cells at a MOI of 200. GroEL, bacterial cytosolic protein; actin, eukaryotic cytosolic protein. Each lane is an independent well from one experiment. (B and D) Quantification of cytosolic effectors FLAG-tagged IpaA (B) and TEM-tagged OspB (D) from experiments depicted in panels A and C, respectively. *, p<0.05; ***, p<0.001; Student’s t-test. (E) Schematic diagram showing differences between induced type 3 mediated secretion from bacteria in liquid media, which results in bacterial effectors in the extracellular medium, and plasma membrane contact-induced type 3 mediated translocation, which results in bacterial effector protein translocation into the host cytosol. (F-G) Effect of CytoD on type 3 secretion of effectors following induction with Congo red. (F) Bacterial supernatant proteins detected in silver-stained gel. Blots are representative of four experiments; two biological replicates were performed in each experiment. (G) Western blots of bacterial supernatants and pellets, representative of three independent experiments. (F-G) IpaA, IpaB, IpaC, IpaD, IpaH’s, and IpgD, type 3 secreted proteins. GroEL, bacterial cytoplasmic protein; SepA and IcsA, type 5 secreted proteins, whose secretion occurs independent of both the T3SS and Congo red. (TIF) [file ppat.1009932.s001.tif]

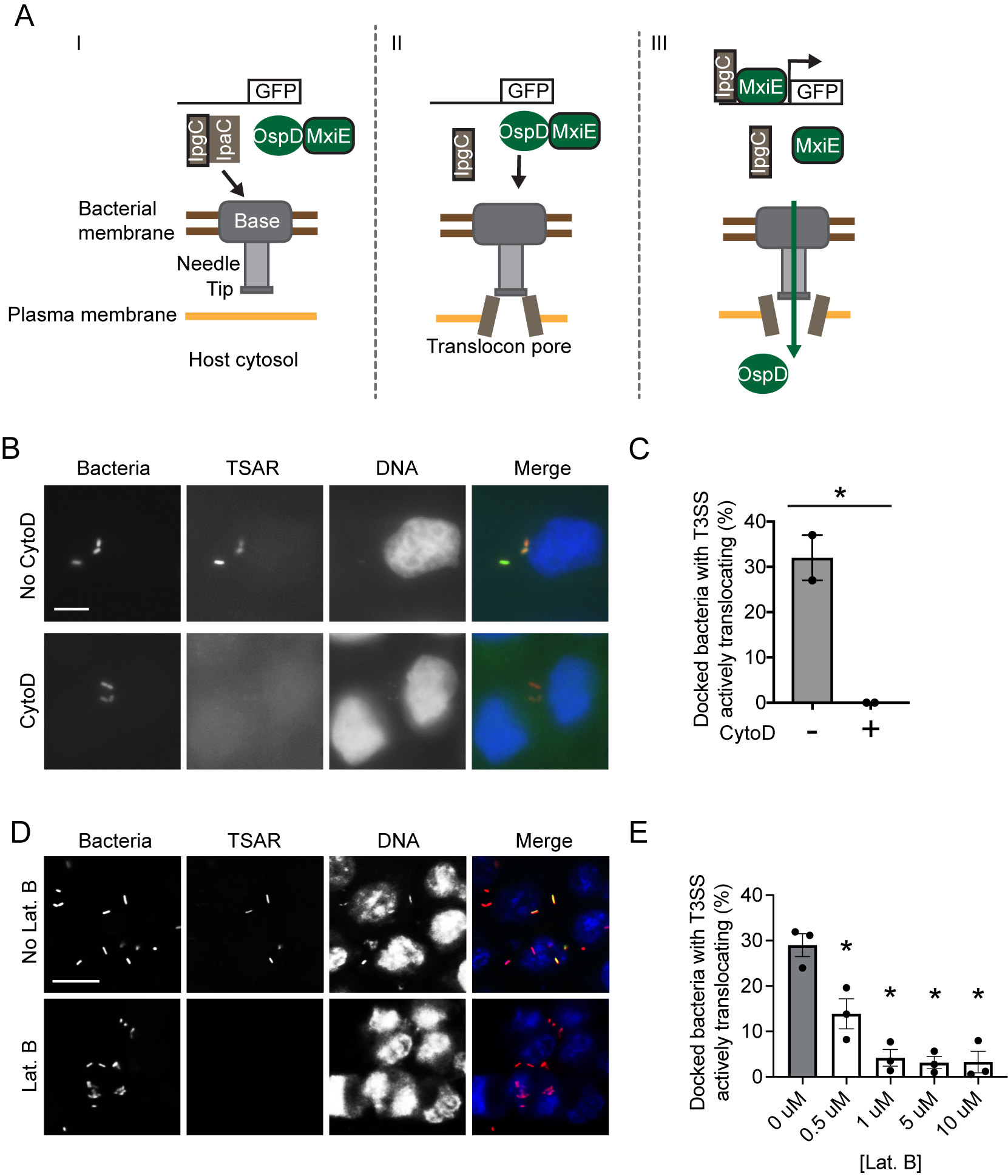

Supplement: S2 Fig — (A) Schematic depiction of OspD-dependent production of GFP by the TSAR reporter. Transient contact with host plasma membrane activates secretion of the translocon pore proteins IpaC and IpaB (IpaB not shown) (I). The secretion of IpaC and IpaB liberates their cognate chaperone, IpgC, and secreted IpaB and IpaC form the translocon pore in the plasma membrane, onto which the bacterium docks (II). OspD translocation liberates its chaperone, MxiE. IpgC binds to and activates MxiE, and IpgC-MxiE functions as a transcriptional activator, inducing the mxiE promoter upstream of gfp (III). HeLa cells infected with S. flexneri carrying TSAR with or without cytoD (B-C) or latrunculin B (D-E) at a MOI of 200. (B and D) Representative fluorescent images of cells treated with or without 5 μm Lat. B. Blue, DNA (Hoechst); red, mCherry (constitutively produced); green, GFP (transcriptionally activated by the secretion of OspD). Scale bar 10 μm (B) or 20 μm (D). (C and E) Percentage of docked bacteria with active secretion in experiments represented in panel B or D. Data points represent independent experiments. *, p<0.05; Student’s t-test. (TIF) [file ppat.1009932.s002.tif]

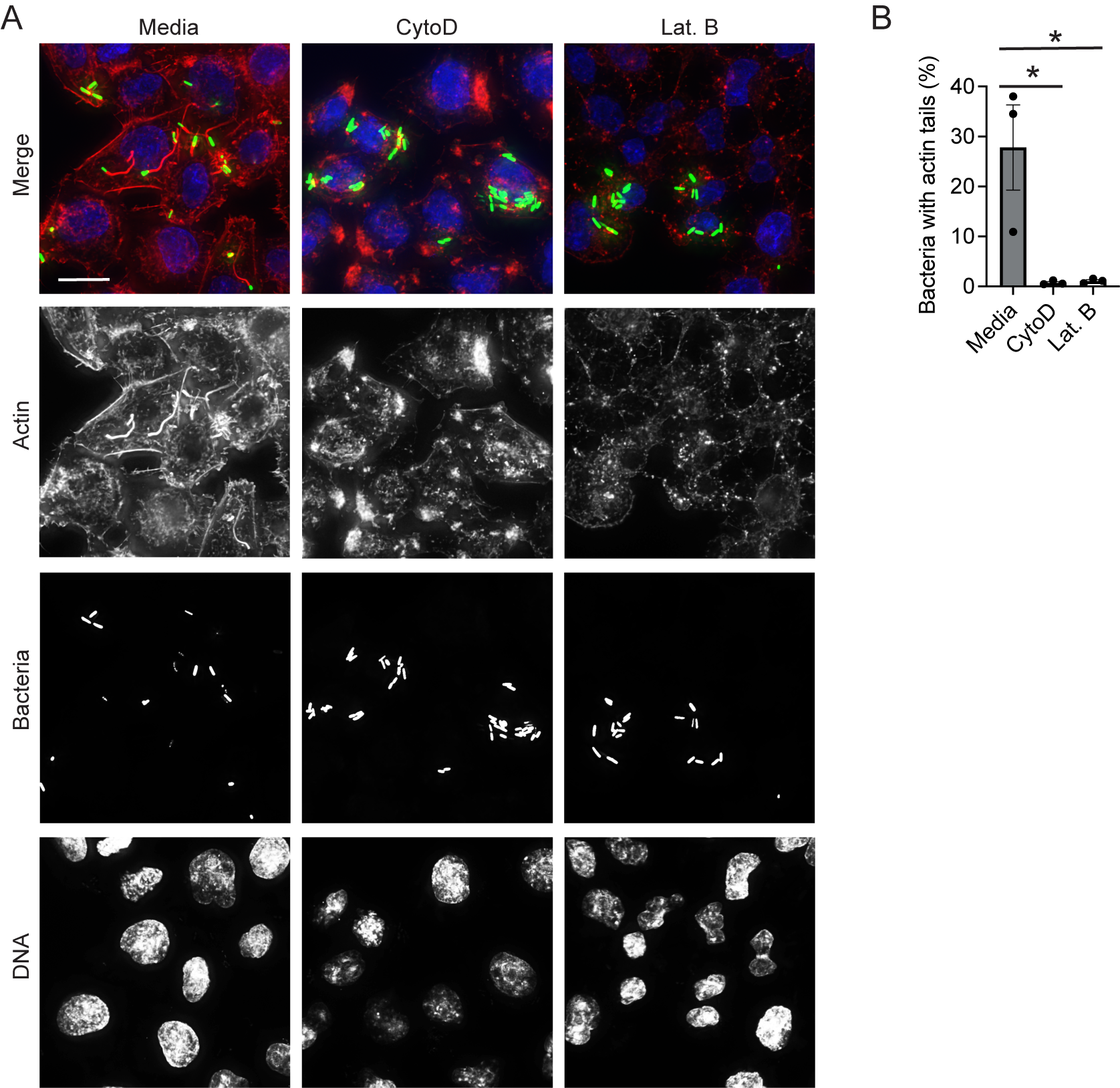

Supplement: S3 Fig — Actin tail formation in HeLa cells infected with S. flexneri at an MOI of 200 in the presence or absence of cytochalasin D or latrunculin B. (A) Representative fluorescent microscopy images. Blue, DNA; green, bacteria; red, actin. Scale bar 20 μm. (B) Quantification of the efficiency of actin tail formation from images presented in panel A. Data are mean ± SEM of three independent experiments. Data points are independent experiments. *, p<0.05, one-way ANOVA with Dunnett’s post hoc test. (TIF) [file ppat.1009932.s003.tif]

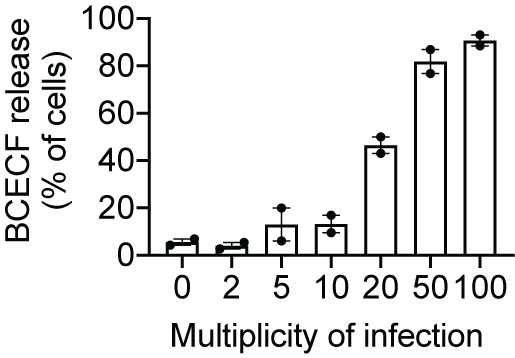

Supplement: S4 Fig — BCECF dye released from HeLa cells infected with E. coli pSfT3SS as a function of multiplicity of infection. Data are mean ± SEM of two independent experiments; data points are independent experiments. (TIF) [file ppat.1009932.s004.tif]

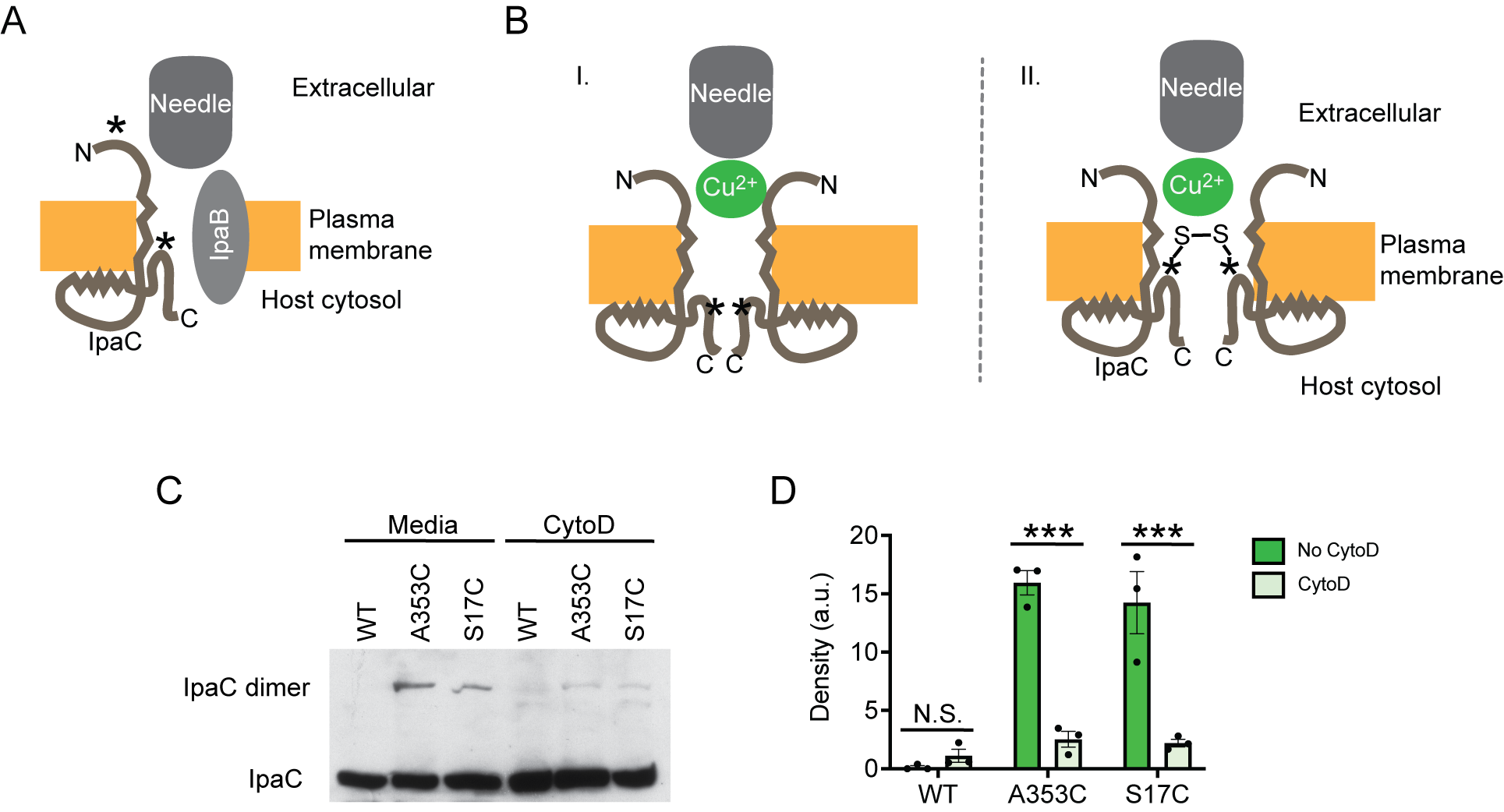

Supplement: S5 Fig — (A) Schematic depiction of the position of S17C and A353C in IpaC indicated by asterisk. (B) Schematic depiction showing that disulfide bonds do not form between adjacent IpaC monomers when A353C is in the cytosol (I), but can form in an intermediary pore complex when an A353C-containing loop of IpaC extends into the lumen of the pore (II). (C-D) Effect of cytoD on the ability of the oxidant copper to induce crosslinking between IpaC monomers at S17C and A353C. HeLa cells were infected at a MOI of 200. (C) Representative western blot. (D) Quantification of crosslinked dimer band density in panel D. Data are the mean ± SEM of three independent experiments. Data points are independent experiments. ***, p<0.001 by two-way ANOVA with Sidak post hoc test. (TIF) [file ppat.1009932.s005.tif]

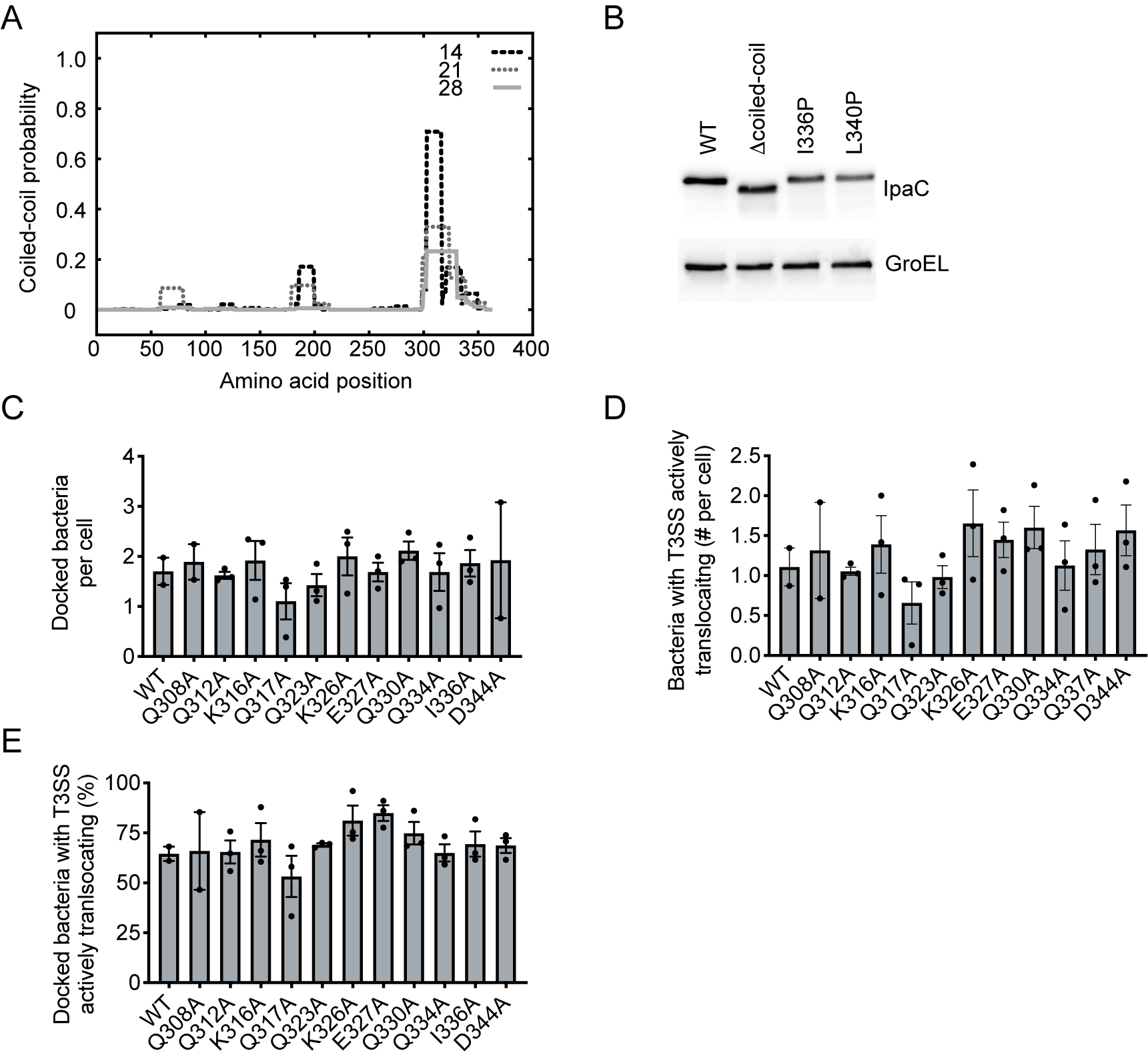

Supplement: S6 Fig — (A) Prediction of coiled-coil domains in IpaC by COILS [55]; 14, 21, and 28 indicate the number of amino acids in each coil. (B) Western blot of IpaC produced by S. flexneri ΔipaC strains induced to produce IpaC alleles or GroEL, a cytoplasmic bacterial protein. (C-E) Docking and translocation into MEFs infected at a MOI of 200 by S. flexneri strains producing indicated IpaC alanine mutant. (C) Docked bacteria per cell at 50 minutes of infection. (D) Number of bacteria with active secretion per cell. (E) Percentage of docked bacteria with active secretion. (C-E) Data are mean ± SEM from two to three independent experiments; data points represent individual experiments. (TIF) [file ppat.1009932.s006.tif]

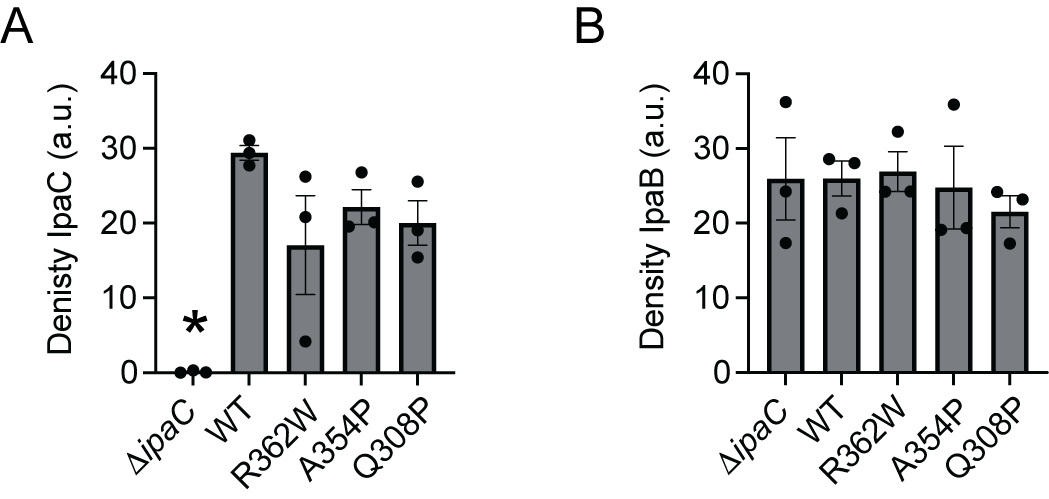

Supplement: S7 Fig — Quantification of western bands of IpaC (A) or IpaB (B) from Fig 5H. Data are mean ± SEM from three independent experiments. *, P<0.05; one-way ANOVA with Dunnett’s post hoc test. (TIF) [file ppat.1009932.s007.tif]

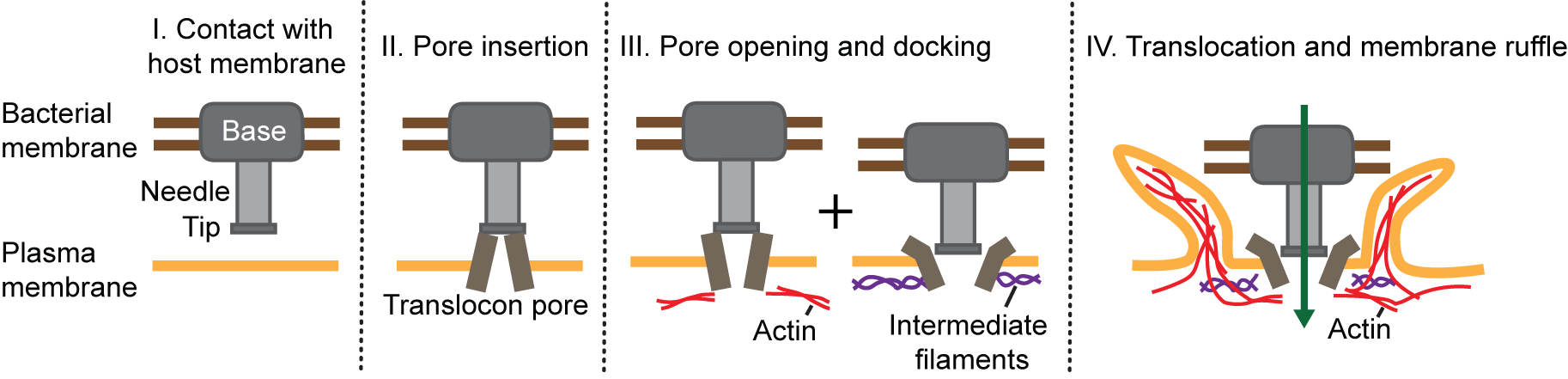

Supplement: S8 Fig — Contact of the T3SS with the host plasma membrane (I) induces the T3SS to deliver the translocon pore proteins into the plasma membrane (II). Actin polymerization opens the pore and the interaction of IpaC with intermediate filaments promotes bacterial docking onto the pore complex (III). Effectors are secreted through the T3SS, and together with IpaC, trigger membrane ruffle formation (IV) and consequent bacterial uptake. (TIF) [file ppat.1009932.s008.tif]
