# Supplementary material for: The type 3 secretion system requires actin polymerization to open translocon pores
Source: PLoS Pathog. 2021 Sep 9;17(9):e1009932. doi: 10.1371/journal.ppat.1009932 (PMC8454972; doi:10.1371/journal.ppat.1009932)
Supplement: Attachment — Submitted filename: Russo et al - July 2021.docx [file ppat.1009932.s010.docx]

This manuscript by Russo et al. describes changes that occur within the IpaC component of the Shigella T3SS translocon brought about by the stimulation of actin polymerization at the site of bacterial attachment. These changes are proposed to be required to permit translocon opening, which allows for the passage of effector proteins into the host cell cytoplasm. Translocon opening correlates with the indirect measurement of a structural or organizational change within the translocon that affects the ability to label introduced Cys residues with PEG-maleimide. The portion of IpaC that is responsible for the opening of the pore is the coiled-coil domain located near the C-terminus of the protein. The coiled-coil domain was then found to not be required for docking (an event that involves IpaC recruitment of intermediate filaments). The authors then state that the actin polymerization-dependent pore opening appears to be distinct from the membrane ruffling events that precede bacterial uptake. This paper offers some rather elegant experiments and provides new insights to events occurring at the host-pathogen interface. While I very much like that paper, there are some things the authors should consider that could help improve it.

Comments:

Related to figure 1, how might secretion into the culture supernatant differ from secretion by internalized (cytoplasmic) bacteria? Is it not possible that the increased secretion seen associated with actin polymerization is due to internalized bacteria that may not have a membrane-associated translocon at the needle tip? Overall, it’s not clear that Figure 1 “shows” that actin polymerization is required for effector translocation.

Page 7, line 116: The authors state that “although actin polymerization had a ‘minor’ effect on the efficiency of bacterial docking”, which appears to be somewhat misleading. If looking at it correctly, Figure 1E seems to show a significant difference of p<0.01 for docking which does not seem to be minor. The language used by the authors would appear to be somewhat biased at times by somewhat overstating the strength of data at times and somewhat understating findings at other times.

The experiments involving the release of BCECF (page 8) provide the first strong evidence that actin polymerization is truly contributing to the opening of translocon pores that are imbedded in the host membrane (rather than there being increased translocation following bacteria entry). This is a very nicely done experiment.

The authors should explain their data in figure 5 a bit more clearly. It’s not clear to me how those data truly show that the actin polymerization that opens the translocon is distinct from that responsible for membrane ruffling. I’m just not following their argument all that well here.

Minor points:

Page 8, line 139: For Figure S3A, it does not appear that there is a Figure S3B, so that should be changed to simply Figure S3.

Page 11, line 194: The authors mention distinct conformational changes, but the use of the term distinct probably is better served to say there are distinct conformation (or organizational) states. “Distinct” could be deleted, however, if the author mean “specific”, they are probably overstating their case since all these measurements are indirect or, at best, only suggestive.
