# Supplementary material for: The type 3 secretion system requires actin polymerization to open translocon pores
Source: PLoS Pathog. 2021 Sep 9;17(9):e1009932. doi: 10.1371/journal.ppat.1009932 (PMC8454972; doi:10.1371/journal.ppat.1009932)
Supplement: Attachment — Submitted filename: PLOSPathogens Response to Reviewers Final.docx [file ppat.1009932.s011.docx]

**We thank the reviewers for their thoughtful criticisms of our manuscript, we feel that the suggestions have much improved the quality of the manuscript. Below, we provide responses to your concerns.**

Editor:

The reviewers agree on the merits of the manuscript, but found issues with the discussion being one-sided and lacking alternative interpretations. This is important since the majority of the conclusions is driven by cytochalasin treatment of cells, which may lead to both direct and indirect alterations in the host cells. These different effects can alter interpretation of the data presented and should be discussed in a more balanced manner.

**We now provide a more balanced and nuanced interpretation and discussion of our results. We also provide new data showing the effects observed on translocation are not limited to cytoD treatment but also occur in response to latrunculin B treatment.**

Minor comments
Legends should include MOI

**MOIs are now included in the figure legends.**


Reviewer's Responses to Questions

**Part I - Summary**
Please use this section to discuss strengths/weaknesses of study, novelty/significance, general execution and scholarship.

Reviewer #1: Russo et al. provide interesting results supporting the idea that host actin polymerization may induce conformational changes in the Shigella translocation pore protein IpaC, thereby opening pores that allow translocation of effector proteins into host cells. Together with previous work on the role of host vimentin proteins, this study suggests that Shigella may manipulate the functions of two different host cytoskeletal elements to promote distinct and separable steps in effector protein translocation. The data in this manuscript suggests that actin polymerization controls the opening of translocon pores, and previously published results show that binding of IpaC to vimentin induces conformational changes in the pore complex that allow stable docking of Shigella.

This is an interesting study that is generally well performed. My main substantive criticism is that the evidence that host actin polymerization is needed to open translocon pores is somewhat weak, in that it depends entirely on a single approach (i.e. cytochalasin D treatment). The effects of cytochalasin D treatment are not confirmed through the use of other methods to inhibit actin polymerization (e.g. treatment with latrunculin A or RNAi-mediated depletion of components of the Arp2/3 complex or other host proteins that promote actin filament assembly). Moreover (and most importantly), the actin polymerization event that is inferred to happen (based on experiments with cytochalasin D) is never directly shown to occur during docking or effector translocation through the use of microscopy-based approaches. As a result, while the data certainly suggest a role for actin polymerization in opening of translocon pores, the work falls short of unequivocally demonstrating that Shigella stimulates actin polymerization to promote effector protein translocation. Perhaps only an intact host actin cytoskeleton (which would be disrupted by cytochalasin D treatment) is needed for translocation, without bacteria directly and locally stimulating actin filament assembly.

Reviewer #2: The findings presented here show that there is an interplay between the host actin cytoskeleton and the Ipa proteins that make up the membrane-embedded translocon pore of the Shigella T3SS. Specific signals elicited by IpaC promote actin polymerization which opens the translocon pore to permit the subsequent passage of effector proteins into the host cytosol. The authors have already attributed a role for intermediate filaments in the docking of the T3SS needle and translocon to the host cell with IpaC also contributing to this process, however, the actin polymerization involved with translocon opening is distinct from that process. Thus, these findings are new and propel the field forward with regard to understanding the molecular events controlling translocation. The manuscript also puts this process into the context of what is known for other T3SS. The experiments described are elegantly designed and the authors are to be credited with the fact that these are not particularly simple experiments to execute. I will also upload my separately written review.

Reviewer #3: NoneEffector secretion by T3SS is triggered by host cell contact, the mechanism by which host cell contact is sensed is unclear but involves the translocon. Russo et al. present evidence that actin polymerization in the host cell is needed for effector secretion to commence. This involves a conformational change in the translocation pore and is distinct from the intermediate filament-mediated docking of the needle-tip to the translocation pore. The experiments are well executed and the data is presented clearly. My primary comments regard the interpretation of the results. Specifically, does actin polymerization really mediate “pore opening” as opposed to a conformational change that “flips the switch” to effector export in the bacterial cytoplasm (presumably by propagating the conformational change from the pore down the needle to the base of the apparatus)?

Add discussion about the possibility that the pore is not opening but undergoing a conformational change.

**Part II – Major Issues: Key Experiments Required for Acceptance**
Please use this section to detail the key new experiments or modifications of existing experiments that should be absolutely required to validate study conclusions.

Generally, there should be no more than 3 such required experiments or major modifications for a "Major Revision" recommendation. If more than 3 experiments are necessary to validate the study conclusions, then you are encouraged to recommend "Reject".

Reviewer #1: My main substantive criticism is that the evidence that host actin polymerization is needed to open translocon pores is somewhat weak, in that it depends entirely on a single approach (i.e. cytochalasin D treatment).

In this regard, the work would be strengthened by the use of other approaches to inhibit actin polymerization (e.g. treatment with latrunculin A and/or RNAi-mediated depletion of components of the Arp2/3 complex or other host proteins that promote actin filament assembly).

**We now provide additional experimental evidence to support our conclusions with cytochalasin D. We show that latrunculin B blocks translocation of effector proteins into HeLa cells using the TSAR reporter (Fig. S2D-E, Lines 131-136).**

The work would be strengthened even more by data that more directly demonstrates that Shigella locally stimulates actin polymerization during docking and/or effector translocation through the use of microscopy-based approaches. However, I recognize that obtaining such data might be quite difficult if these localized actin polymerization events are weak, highly transient, and/or masked by actin polymerization in ruffles. If experiments to directly detect actin polymerization during docking/translocation cannot be performed because of these technical issues, it would be useful for the authors to include a statement in the discussion section that explains the extent to which their data supports the idea that Shigella induces host actin polymerization to promote opening of translocon pores versus the possibility that pore opening instead simply requires an intact actin cytoskeleton that is not actively manipulated by Shigella.

**We now provide additional discussion indicating that an intact cytoskeleton may be sufficient to enable translocation and pore formation (Lines 373-375).**

Reviewer #2: No additional experiments are needed.

Reviewer #3: none

**Part III – Minor Issues: Editorial and Data Presentation Modifications**
Please use this section for editorial suggestions as well as relatively minor modifications of existing data that would enhance clarity.

Reviewer #1: • Abstract: ‘An IpaC mutant is identified that shows…’. It seems incorrect to state that an IpaC mutant ‘shows’, since it is really the results obtained with this mutant that show something. I would be inclined to change this sentence to something like ‘Results from experiments involving an IpaC mutant defective in … show that . ‘

**Changed as recommended by the reviewer (Line 32).**

• Page 4, first paragraph, lines 48-51. References are needed to support these statements.

**References have been added to support these statements (Lines 48-51).**

• Page 6 and Fig. 1. ‘Actin polymerization is required for type 3 effector protein translocation. What is the evidence that cytochalasin D treatment inhibits actin polymerization under the conditions used in these experiments? Are the levels and/or distribution of F-actin altered by cytochalasin D treatment in these studies? Are similar results obtained by other inhibitors of actin polymerization such as latrunculin A or by genetic inhibition of actin polymerization (e.g. siRNA-mediated depletion of components of the Arp2/3 complex)?

**We now provide data that the concentrations of cytoD and latrunculin B used in our experiments is effective at disrupting the actin cytoskeleton. To do so, we examined the ability of *S. flexneri* to nucleate actin polymerization to form the actin tails that are required for its motility in cells. We show both chemicals significantly impair actin tail formation (Fig. S3, Lines 136-139). Additionally, the actin cytoskeleton following treatment by either chemical appears disrupted compared to untreated cells (Fig. S3A).**

**We now provide additional experimental evidence to support our conclusions with cytochalasin D. We show that latrunculin B blocks translocation of effector proteins into HeLa cells using the TSAR reporter (Fig. S2D-E, Lines 131-136).**

• Fig. 1A. IpaC should be labeled in this diagram. On page 5, it is stated that two proteins comprise the translocation pore in bacterial type III secretion systems. What protein apart from IpaC comprises the translocation pore for Shigella? Is this other protein depicted in Figure 1AI, or is only IpaC shown?

**For *Shigella,* IpaC and IpaB are required to form the translocon pore. The stoichiometry and arrangement of these two molecules is unknown. Based on the reviewer’s suggestions, we further clarified the location of IpaC and the presence of IpaB in the pore for the schematic presented in the figure 1.**

• Figure 1 parts E and F. ANOVA and a post-test should be used for statistical analysis. T-tests should be used only for analysis of two data sets (Olsen, 2014. Infect. Immun. 82: 916-020.

**For Fig. 1E and F, a two-way ANOVA with a Tukey *post hoc* test was performed and is indicated in the figure legend. The figure legend was reorganized to clarify which statistical tests were used.**

• Figure S1, parts F and G. Duplicate samples seem to be loaded on these gels, and it would be good to explicitly state that these are technical replicates.

**As recommended by the reviewer, we have now indicated that the western blot in figure S1F display biological replicates from a representative experiment.**

**Fig. S1G, does not display duplicate samples. Rather, combinations of cytoD and Congo red are displayed from the supernatant and pellet.**

• Figure 2E. Why were IpaB levels in the membrane not quantified?

**In the initial manuscript, we focused on IpaC, as its insertion into the membrane is dependent on IpaB insertion in the membrane, and it was previously described to have a role in altering actin dynamics. In the revised manuscript, we now also include densitometry analysis of IpaB in the membrane (Fig. 2F). Similar to IpaC, CytoD did not alter the abundance of IpaB in the membrane.**

• Methods. The correct spelling is ‘scraped’, and not ‘scrapped’.

**Thank you for picking up this error. Corrected as recommended.**

• Line 158. Maleimide is misspelled.

**Thank you for picking up this error. Corrected as recommended.**

• Legend to Figure 3 line 705. Should ‘A353’ really be ‘A358’?

**Thank you for picking up this error. Yes, this is now changed to 358.**

• Figure 3A,C,D. It seems strange that the PEG-maleimide accessibility of A358, which is depicted as inside the host cytosol (or close to it) is not affected by interaction with vimentins. Isn’t the IpaCR362W mutant supposed to be defective in activating the translocon pore? If so, then how can PEG-maleimide get into the host cytosol to label A358 in IpaC?

**We previously showed that vimentin does not alter the pore opening, since BCECF release occurs efficiently in the presence and absence of intermediate filaments (Russo, *et. al.* Nat. Micro 2016). This phenotype also occurs in cells containing intermediate filaments with the R362W mutant, which is unable to interact with intermediate filaments; IpaC R362W inserts into the membrane, and translocon pores that contain it are open (at least when actin polymerization occurs) (Russo, *et. al.* Nat. Micro 2016). We demonstrate the ability of IpaC R362W to form open pores in this manuscript (Fig. 4C-D and Fig. 5F-G). The IpaC R362W mutant is defective in its ability to support docking onto the pore (~20% of the level of docking supported by WT IpaC), and consequently is associated with reduced activation of translocation.**

**Since the pore is open whether or not IpaC interacts with intermediate filaments, the C-terminal region of IpaC is accessible. We previously showed that in contrast to A358, whose accessibility is not altered in the absence of intermediate filaments, other C-terminal residues, such as S349, have diminished accessibility in the absence of intermediate filaments (Russo *et. al.* Plos Pathogens 2019). The findings presented here are consistent with our previous observations about these particular residues and the role of intermediate filaments.**

**In the revised manuscript, we now clarify these points (Lines 192-193).**

• Legend to Figure 3D. Some of the data are apparently from only two experiments. However, statistical analysis cannot (or at least should not) be performed on only two data sets. In my view, it is essential to perform all experiments at least three times and perform statistical analysis on the resulting three data sets to confirm significance and reproducibility.

**In Figure 3, the only experiment that was performed only twice was the accessibility of IpaC A358C; accessibility of all other residues was tested three times. We have previously published that there is not a difference in the accessibility of A358C in WT IpaC or IpaC R362W (Russo *et. al.* Plos Pathogens 2019); thus, the data shown here are reproducible across multiple studies. Here, we additionally show that there is less accessibility of A358C in the presence of CytoD. We feel further experiments would not change our overall conclusion that the positioning of IpaC residues/molecules is altered by CytoD treatment and have chosen not to perform additional experiments. To alleviate the reviewers concern, in the revised manuscript, we removed from the figure the statistical analysis relating to this residue.**

• Since IpaC is known to contribute to actin polymerization, could the lack of effect of cytochalasin D treatment on PEG-maleimide labeling of IpaC.S17C be due to a defect in the ability of IpaC to promote actin polymerization? In this case, I’m not referring to actin polymerization in ruffles, but rather at sites of translocon opening.

**We are not sure about what the reviewer is asking about specifically in reference to IpaC-S17C labeling by PEG maleimide, as the PEG labelling of this residue is significantly affected by CytoD treatment (Fig. 3). What we can say is that we do not believe that IpaC S17C is defective in functional activities of IpaC. We have characterized IpaC S17C and the other IpaC cysteine mutants extensively, showing that they form translocon pores, support translocation, and enable invasion of cells as efficiently as wild-type IpaC (Russo et. al. mBio 2019). Although we have not specifically tested for the actin phenotype presented here, we think it is unlikely that it is significantly altered for IpaC S17C, given that we have not observed an impact of this and these other mutations on any other aspect of IpaC function during *S. flexneri* infection.**

• Line 202 and legend to Figure S4. Is ‘A353C’ correct, or do the authors mean ‘A358C’?

**For Fig. S4 (now S5) and line 202, we used A353C.**

• Figure S4. It would be nice to have a control involving a cysteine mutation around the N-terminus of IpaC. If I am understanding this approach correctly, then such a mutant protein would be cross-linked in a manner that is unaffected by cytochalasin D treatment, since the cysteine residue would not be in the cytosol of the host cell.

**We thank the reviewer for this suggestion. S17C is an N-terminal residue. The accessibility of this residue is significantly altered by the presence of cytoD (Fig. 3) and the lack of crosslinking in the presence of cytochalasin D (Fig. S4) is consistent with the lack of accessibility of this residue by PEG-maleimide under these conditions. It is unclear to us why this is the case. We speculate that the lack of accessibility may be due to interactions of the N-terminal region of IpaC with the needle. These findings nevertheless shows that actin polymerization or some actin dependent process is necessary to transition IpaC into a translocation competent position within the pore, and thus, these results support the major findings of this manuscript.**

• Figure 4. Are the IpaC coiled coil domain mutants expressed at levels similar to that of wild-type IpaC?

**Yes, we have included a representative western blot (Fig. S6B).**

• Page 14, lines 268-269. The reader should be referred to Figure 5F,G.

**We have made this change as recommended.**

• Figure 5H. Quantified Western blotting data from three experiments and resulting statistical analysis should be performed. By eye, IpaB and IpaC translocation seem to be slightly reduced for the Q308P mutant.

**Based on the reviewer’s recommendation, we now provide quantification of these blots (Fig. S7). Please note, Fig. 5H does not present data on *translocation* of IpaB or IpaC per se; rather, these data show the amount of protein delivered into the plasma membrane of the host cell.**

Reviewer #2: A couple of minor comments are included in the uploaded review. **(See below)**

Reviewer #3: BCECF release. I always thought that the actin polymerization in the absence of effector translocation dissociated the pore from the needle, allowing efflux. It’s less of an “open pore” and more of a dissociation of the pore from the needle assay then? (e.g. Viboud and Bliska 2001 EMBO J)

**We don’t have evidence supporting a dissociation event, but in our assays, we cannot rule out that one is occurring.**

**As the reviewer correctly notes, Viboud and Bliska (2001 EMBO J) speculate in the paper’s Discussion that actin polymerization enables BCECF release (pore formation) by enabling a significant amount of membrane movement in the vicinity of the bacteria, which potentially dissociates the needle from the pore. However, there is no direct evidence that this is occurring; rather, it is suggested as a potential explanation for how actin polymerization may enable dye release.**

**For the following reasons, we feel it unlikely that actin polymerization in the absence of effector translocation leads to dissociation of the pore from the needle. Mejia, Bliska, and Viboud (Plos Path 2009) show that for Yersinia, interaction with β1 integrin is necessary for pore insertion and effector translocation and that this is due to enhanced actin polymerization. They also show that cell-associated Yersinia have actin halos, demonstrating an intimate association of the bacteria with the cell. They further discuss how greater amounts of actin polymerization are associated with more efficient translocation. The observation that increased actin polymerization would be associated with greater translocation leads us to speculate that it would be unlikely that the actin polymerization would be associated with a dissociation of the needle from the pore. One potential caveat to our interpretation of these data is that rather than focusing on actin polymerization in the absence of T3SS effectors, these experiments were carried out with bacteria containing T3SS effectors.**

**For *Shigella*, our previous data show that pore opening and docking (the stable association of the needle with the pore) are not directly linked. Under conditions in which docking is weak (the absence of intermediate filaments such as vimentin), BCECF release occurs at a rate that is similar to conditions in which the needle stably maintains the association with the pore (Russo et. al. Nat. Micro 2016). Whether in our experimental conditions BCECF release occurs only from pores that have dissociated from the needle is unknown. Consistent with our data indicating that BCECF release is not linked to association of the needle with the pore, our previous data demonstrate that docking does not restrict access to the interior of the pore. The accessibility of PEG-maleimide to some residues in the interior of the pore, such as A358C (also presented in Fig. 3), is similar for pores that do or do not maintain needle interactions (Russo et. al. Plos Path 2019). In addition, the *E. coli* strain we are using expresses four of the >30 effectors (Reeves et. al. ACS Synth Biol. 2015), so it is not completely lacking effector translocation, and it invades cells at levels similar to those of wild-type *Shigella* (Du et. al. PNAS 2015). Altogether, we believe that our previous data and those reported for Yersinia argue against a dissociation event.**

In this context, in Fig. 3, why is labeling in the presence of cytoD generally much weaker than without, even for extracellular residues, such as S17C. If BCECF release is due to actin polymerization-dependent dissociation of the pore from the needle-tip, could the lack of accessibility in the presence of cytoD mean that the reagent cannot access the cysteines in the assembled needle-tip/pore complex? The same could be going on with the reduced disulfide bond formation at position S17 in the presence of copper (Fig. S4C/D).

**We thank the reviewer for their suggestion. In the revised manuscript, we provide additional discussion that discusses the observed reduction in labeling/crosslinking of the N-terminal residues (lines 363-377).**

Notably, to argue against my own hypothesis. The reduction in docking upon cytoD treatment (Fig. 1E) would argue against stabilization of the translocon. However, in general, I find that the data could be argued both ways, and perhaps the authors could spend a bit more time on alternative explanations for their data? To my mind, the question of whether triggering of effector secretion is the result of opening of the translocation pore, or a conformational change in the translocation pore that results in a change in export specificity is not resolved.

**We agree with the reviewer that multiple outcomes are possible with our current understanding of the pore. In the revised manuscript, we now provide additional discussion about the possible role of actin polymerization in altering the dynamics at the cell surface and of the pore with the needle (Discussion, lines 363-377).**

Another point that the authors could mention is that the notion of a conformational change in the translocation pore triggers effector secretion has also been found in Pseudomonas aeruginosa (Armentrout 2016 PLoS Pathogens). Notably, the minor translocator, PopD, which is analogous to IpaC in that system, was implicated in the signal transduction process. It is interesting that, while these systems are different (PopD topology differs from IpaC, cytoD does not interefere with translocation, etc), the overall mechanism for engaging the T3SS seems to be conserved (at least the 10000ft view of it).

**We thank the reviewer for the suggestion; in the revised manuscript, we reference these findings in the Discussion (line 384-386).**

Fig. 5D-E – If I read the literature correctly, then membrane ruffling is promoted not only by IpaC, but also by effector proteins that are injected into the host cell. As a result, you don’t really know if these mutants are defective in IpaC-mediated ruffle-formation, since the phenotype could be indirect, a reflection of the defect in effector injection. Hemolysis, likely a direct function of the translocation pore, is less severely affected by the mutations than activation of the TSAR reporter or membrane ruffling, which requires the additional step of activating effector injection (and efficient effector delivery). Which means multiple steps could be compromised here. In general, I feel that the data in Fig. 5 could benefit from a more nuanced analysis of the potential pitfalls in their interpretation.

**We thank the reviewer for their suggestion and now provide a more nuanced interpretation of the data in Figure 5D-E in the Results section (lines 284-291).**

 Reviewer #2. This manuscript by Russo et al. describes changes that occur within the IpaC component of the Shigella T3SS translocon brought about by the stimulation of actin polymerization at the site of bacterial attachment. These changes are proposed to be required to permit translocon opening, which allows for the passage of effector proteins into the host cell cytoplasm. Translocon opening correlates with the indirect measurement of a structural or organizational change within the translocon that affects the ability to label introduced Cys residues with PEG-maleimide. The portion of IpaC that is responsible for the opening of the pore is the coiled-coil domain located near the C-terminus of the protein. The coiled-coil domain was then found to not be required for docking (an event that involves IpaC recruitment of intermediate filaments). The authors then state that the actin polymerization-dependent pore opening appears to be distinct from the membrane ruffling events that precede bacterial uptake. This paper offers some rather elegant experiments and provides new insights to events occurring at the host-pathogen interface. While I very much like that paper, there are some things the authors should consider that could help improve it.

Comments:

Related to figure 1, how might secretion into the culture supernatant differ from secretion by internalized (cytoplasmic) bacteria? Is it not possible that the increased secretion seen associated with actin polymerization is due to internalized bacteria that may not have a membrane-associated translocon at the needle tip? Overall, it’s not clear that Figure 1 “shows” that actin polymerization is required for effector translocation.

**We agree with the reviewer that it is possible that internalized bacteria contribute to the pool of bacterial effectors detected in the cell cytosol. In the revised manuscript, we modify our language in discussing these results to reflect the reviewer’s concern (Lines 86-92).**

**We nevertheless believe that whether or not the effectors detected in the cytosol represent outright translocation or effector secretion, this particular data set demonstrates that cytoD is blocking an upstream process preventing either effector translocation and/or bacterial entry into the cell. Furthermore, docked bacteria in the presence of CytoD do not activate TSAR, supporting the conclusion that the differences in translocated effector are likely due at least in part to decreased effector delivery at docking.**

Page 7, line 116: The authors state that “although actin polymerization had a ‘minor’ effect on the efficiency of bacterial docking”, which appears to be somewhat misleading. If looking at it correctly, Figure 1E seems to show a significant difference of p<0.01 for docking which does not seem to be minor. The language used by the authors would appear to be somewhat biased at times by somewhat overstating the strength of data at times and somewhat understating findings at other times.

**We thank the reviewer for their suggestion and, in the revised manuscript, we now reference the degree to which cytoD impairs docking (lines 117-119).**

The experiments involving the release of BCECF (page 8) provide the first strong evidence that actin polymerization is truly contributing to the opening of translocon pores that are imbedded in the host membrane (rather than there being increased translocation following bacteria entry). This is a very nicely done experiment.

**We thank the reviewer for their comment!**

The authors should explain their data in figure 5 a bit more clearly. It’s not clear to me how those data truly show that the actin polymerization that opens the translocon is distinct from that responsible for membrane ruffling. I’m just not following their argument all that well here.

**We thank the reviewer for their suggestion. In the revised manuscript, we provide clearer rationale and interpretation of the data presented in Figure 5 (lines 278-310).**

Minor points:

Page 8, line 139: For Figure S3A, it does not appear that there is a Figure S3B, so that should be changed to simply Figure S3.

**This is now changed as recommended (now Fig. S4 [previously S3]).**

Page 11, line 194: The authors mention distinct conformational changes, but the use of the term distinct probably is better served to say there are distinct conformation (or organizational) states. “Distinct” could be deleted, however, if the authors mean “specific”, they are probably overstating their case since all these measurements are indirect or, at best, only suggestive.

**In the revised manuscript, we have changed the wording as recommended by the reviewer (lines 207-208, 225-226, 380).**
